# Cerebrovascular Accident: An Initial Presentation of Atrial Myxoma in a Young Female

**DOI:** 10.7759/cureus.33529

**Published:** 2023-01-09

**Authors:** Nkemputaife P Onyechi, Henry C Egbuchiem, James P Cappello

**Affiliations:** 1 Internal Medicine, University Hospitals Geauga Medical Center, Cleveland, USA

**Keywords:** echocardiogram, neurovascular deficits, emboli, left atrium, cva, myxoma

## Abstract

Myxomas make up roughly half of all primary cardiac tumors, with three-quarters originating in the left atrial cavity and one-quarter in the right. These tumors can occur sporadically or in families. Atrial myxomas are classified as severe based on factors such as their size, the patient's age and gender, and the tumor's proclivity to embolize or occlude coronary vessels. The case of a young female with an unusual presentation of left atrial myxoma and a new neurovascular deficit is discussed.

## Introduction

Cardiac myxoma accounts for 50-80% of primary heart tumors globally, and is important, though relatively uncommon, cause of cerebrovascular diseases [1]. The neurological complication occurs in more than 50% of myxoma patients and can be the initial manifestation. The most frequent neurological complication of cardiac myxoma is cerebral embolism [2]. Cardiac myxomas are the most common primary cardiac tumor in adults, representing as many as 83% of all primary tumors of the heart. Myxomas are particularly frequent from the third to the sixth decades of life and show a 2:1 female predominance [2,3]. Most myxomas occur in the left atrium (83-88%) [3]. We present a rare case of a young female presenting with a cerebrovascular accident (CVA) as the first symptom of an underlying atrial myxoma. We aim to demonstrate that, while myxoma embolization is more common in men [4], it is important to consider in women with new-onset cerebrovascular accidents.

## Case presentation

**Figure 1:**
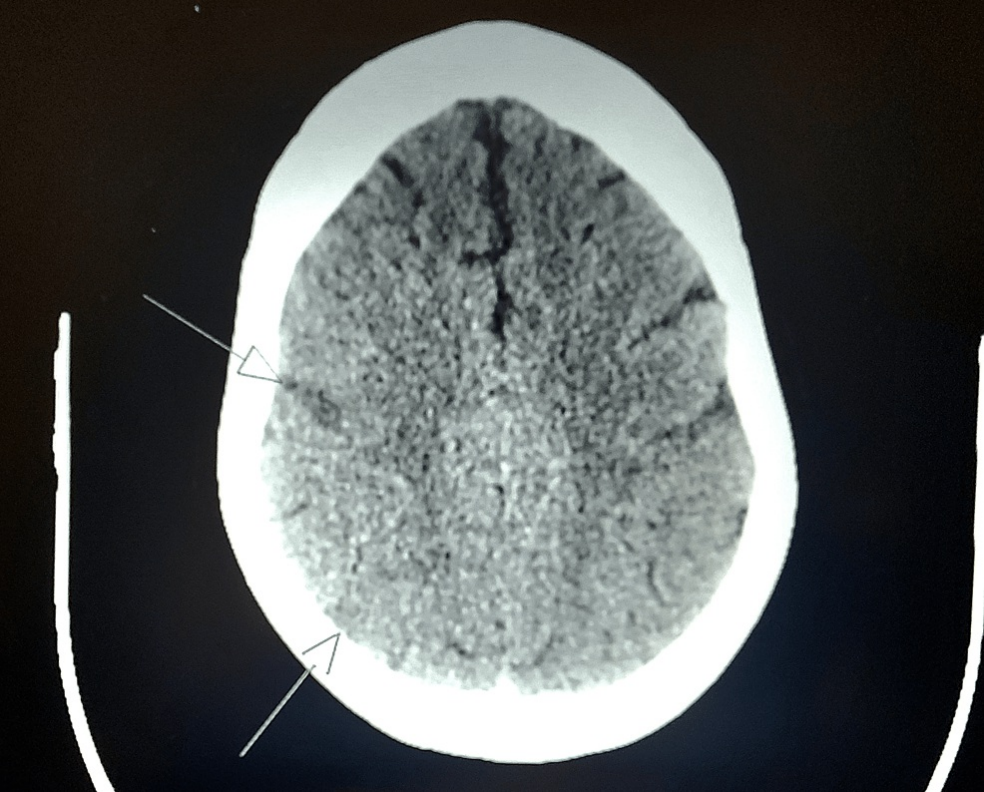
CT brain showing acute infarct in the right frontal and right parietal lobes. Arrows demonstrate two small hypodense areas indicative of evolving small acute infarcts in the right frontal and parietal lobes.

**Figure 2:**
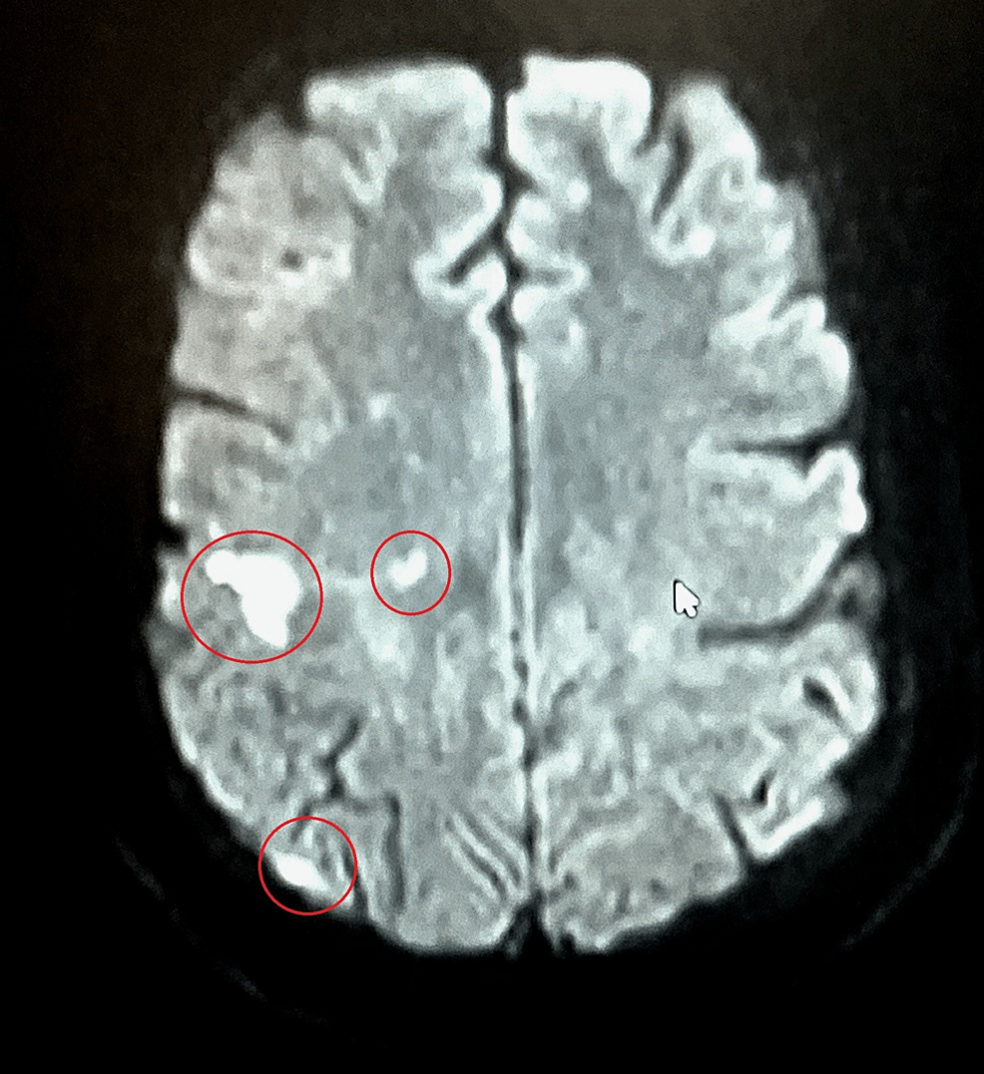
MRI showing scattered multifocal lesions of acute and subacute infarctions. MRI demonstrating small scattered foci of acute to early subacute infarction within the cerebral hemispheres bilaterally right greater than left. The largest of the scattered foci of abnormal diffusion restriction are identified along the posterior right frontal lobe and right parietal lobe.

**Figure 3:**
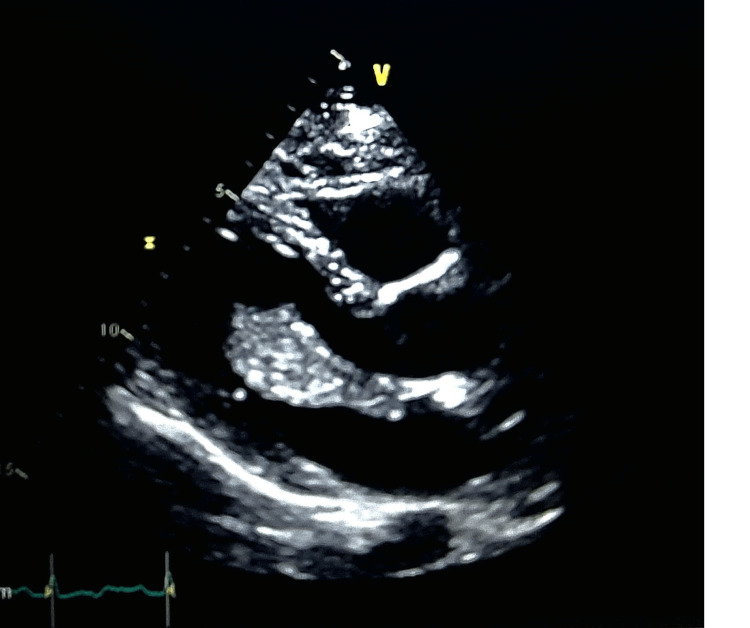
Echocardiogram showing a myxoma in the left atrium. Echocardiogram demonstrating a mass noted in the left atrium. The mass measures approximately 35 mm by 35 mm. The left atrial mass is suggestive of a myxoma.

**Figure 4:**
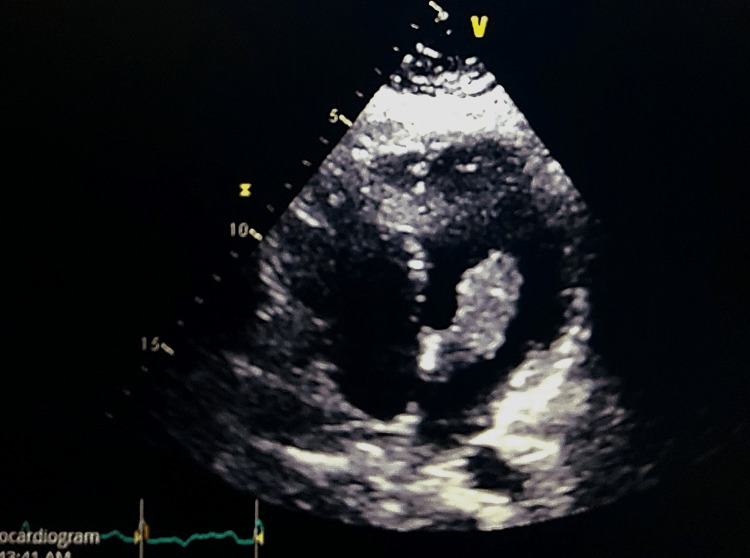
Another view of myxoma in the left atrium.

**Figure 5:**
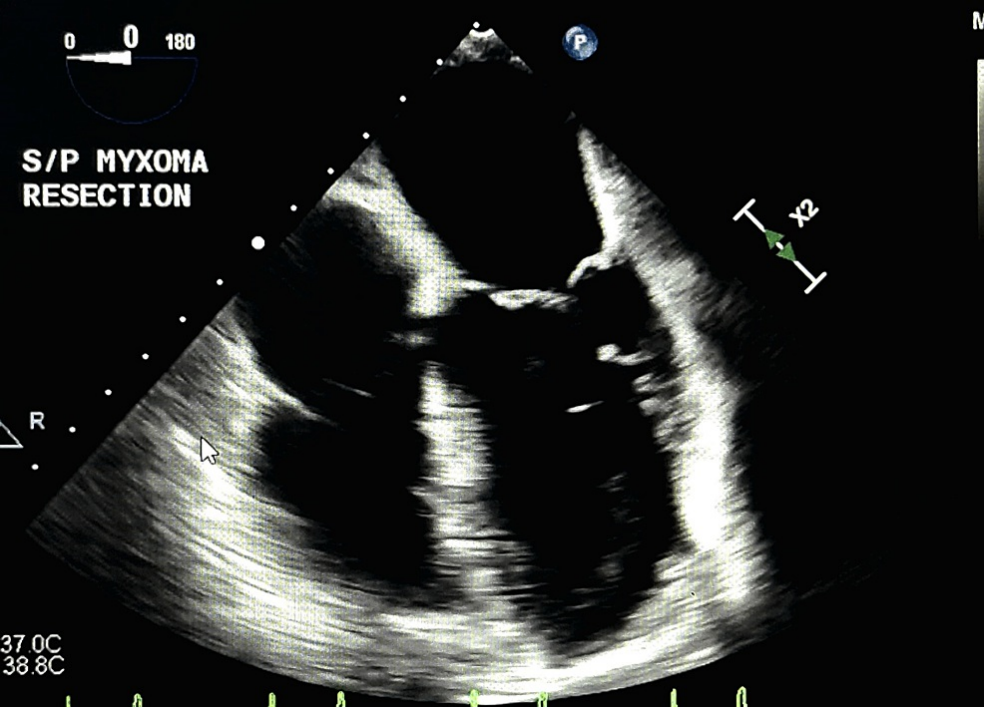
Echocardiogram showing a four-chamber view post-resection of myxoma.

The patient is a 35-year-old female with a history of fibromyalgia and Charcot-Marie-tooth disease who presented to the emergency department with complaints of a 10-hour history of left-sided facial drop and left-hand numbness. Facial droop and hand numbness began at the same time but progressively worsened. She denied dizziness, loss of consciousness, history of falls and head trauma, changes in vision and speech, or difficulty swallowing. She also denied seizures, use of oral contraceptive pills, or a family history of CVA and clot formation. She admitted to occasional marijuana use, smoking a pack of cigarettes per day for 15 years, and drinking five alcoholic drinks per day, with her last drink four days before the presentation. The surgical history was significant for a cesarean section 11 years prior. There was no history of allergies. Vital signs were notable for tachycardia, with a heart rate of 123 bpm and blood pressure of 138/97 mmHg. Physical examination revealed normal cardiovascular and pulmonary examinations, but was positive for left-sided facial drop and 3/5 weakness in the left upper extremity. Laboratory results were hemoglobin 17.9 and negative troponin elevation. Blood chemistries were glucose of 138, with slightly deranged liver enzymes such as aspartate transaminase 67, alanine transaminase 63, and alkaline phosphatase 130. Urine analysis showed the presence of blood and traces of bilirubin, while the urine toxicology test was positive for cannabinoids. A chest x-ray showed resolving pulmonary edema and left basilar atelectasis. The EKG done was unremarkable. A contrast-free CT scan of the brain revealed small hypodense areas of acute and subacute infarcts in the anterior right frontal and parietal lobes (Figure 1). MRI showed small scattered multifocal lesions of acute and subacute infarctions within bilateral cerebral hemispheres and the right parietal lobe (Figure 2). The echocardiogram showed a preserved ejection fraction (EF) of 60-65% with a large mass in the left atrium measuring 35 × 35 mm, suggestive of a myxoma (Figures 3, 4). The diagnosis of lower extremity embolization was ruled out with the use of lower extremity angiography. Since the patient was outside the window for thrombolytics, she was started on dual antiplatelet therapy. The patient subsequently had a median sternotomy for resection of the left atrial myxoma. Post-surgery echo was negative for myxoma (Figure 5). Neurological deficits continued to improve in-patient and were totally resolved at the time of discharge. The excised mass was confirmed by the pathologist to be a myxoma. 

## Discussion

In a study by the Mayo Clinic, of 74 patients diagnosed with atrial myxoma, 12% had neurological complications, and only seven patients in the cohort had neurological symptoms as the initial manifestations of atrial myxoma [3]. Although atrial myxomas are more common in women, females do not usually present with distant clot formations originating from myxomas; conversely, men are more likely to have systemic embolization. It is also important to note that the size of the myxoma affects the embolization rates. In other words, the smaller the myxoma, the more likely it would embolize. A literature review reveals that any size smaller than or equal to 4.5 cm has a higher propensity for systemic embolization. Cardiac myxomas may cause symptoms either because of intracardiac obstruction, systemic embolism of tumor fragments, or constitutional symptoms due to unclear mechanisms. The most feared consequences of cardiac myxomas are preoperative systemic embolism and myxoma recurrence after surgical resection [4]. Of note, our patient had a myxoma dimension of 35 mm × 35 mm, thereby increasing her chance of embolization.

The mean age of atrial myxoma is 56 years for sporadic cases and 25 years for familial cases. The mean age at presentation was 37.1 years. Dyspnea was the most common symptom. Embolism was found in 9% of patients and systemic symptoms in 20% of patients [5]. The left atrium is the preferable location, probably constituting up to 60-80% of all myxomas, and the remaining cases of myxomas can occur in the right atrium, right ventricle, or left ventricle and even some are bi-atrial [6]. Our patient was 35 years old without any cardiovascular symptoms and had no family history of intracardiac tumors. It also appears that her first presentation was a CVA. In addition to the relative rarity of atrial myxoma, the uncommon nature of embolization in females and the predisposition of the tumors in middle-aged or elderly adults make it essential to have a high index of suspicion when caring for females who develop cerebrovascular accidents. It is crucial to include this as a differential because if these myxomas are not recognized early, they can lead to tremendous morbidity and mortality, especially in patients who do not present with cardiovascular symptoms or risk factors.

## Conclusions

Although it is rare for an atrial myxoma to cause embolism in women since embolization is more common in men. In a young female with a new CVA, one should be aware that atrial myxoma could be a potential cause. Smaller myxomas are more likely to cause an embolus (our patient's thrombus size is 35 × 35 mm; any size less than 4.5 cm increases embolization tendencies).
